# 성인 혈우병 환자와 운동: 체계적 문헌고찰과 메타분석

**DOI:** 10.7586/jkbns.23.022

**Published:** 2024-02-28

**Authors:** Doo Young Kim, Mi Yang Jeon, Young Eun, Da In Jeong

**Affiliations:** 1 College of Nursing, Gyeongsang National University, Jinju, Korea; 1 경상국립대학교 간호대학; 2 College of Nursing, Gerontological Health Research Center in Institute of Health Science, Gyeongsang National University, Jinju, Korea; 2 경상국립대학교 간호대학·건강과학연구원 노인건강연구센터; 3 College of Nursing, University of Illinois, Chicago, USA; 3 일리노이주립대 간호대학

**Keywords:** Exercise, Hemophilia, Joints, Pain, Quality of life, 운동, 혈우병, 관절, 통증, 삶의 질

## Abstract

**Purpose:**

This study aimed to evaluate the effectiveness of exercise in patients with hemophilia.

**Methods:**

We carried out a systematic review and meta-analysis in accordance with the Preferred Reporting Items for Systematic Review and Meta-analysis (PRISMA). A literature search was conducted of published randomized controlled trials for exercise interventions from January 1, 2014 to March 15, 2023. To estimate the size of the effects of exercise, a meta-analysis was performed using the R package “meta.”

**Results:**

Five databases were searched to obtain articles published in Korean or English. Of 1,150 articles reviewed, 13 were included in the systematic review and 9 in the meta-analysis. The risk of bias was assessed using RoB 2.0. The overall effect sizes of exercise interventions, calculated as the standardized mean difference, were -0.11 (95% confidence interval [CI] = -1.41 to –1.20) for pain, -2.13 (95% CI = -3.33 to -0.93) for joint health, 9.96 (95% CI = 7.51 to 12.28) for physical activity, and 0.59 (95% CI = -0.39 to -1.56) for quality of life.

**Conclusion:**

These findings suggest that exercise is useful for improving the joint health and physical activity of patients with hemophilia. Thus, it is necessary to develop and apply exercise interventions for patients with hemophilia to reduce their pain and improve their quality of life.

## 서론

### 1. 연구의 필요성

혈우병은 선천적으로 혈액응고 인자 Ⅷ, Ⅸ이 결핍되어 나타나는 유전적 응고장애 질환이다. 과거에는 자연 출혈로 인해 혈우병 환자들의 생존 기간이 짧았다. 그러나 과학과 의학 기술이 지속적으로 발전하면서 새로운 응고인자제제가 개발되고 있다. 세계보건기구가 새롭게 개발되는 응고인자제제를 자연 출혈을 예방하는 표준 치료법으로 인정하고 이를 권장하면서 혈우병 환자의 기대 수명이 약 70세까지 연장되었다[1]. 혈우병 환자들은 혈액응고 인자 Ⅷ, Ⅸ의 결핍[2]과 미세혈관 내피세포의 기능부전으로 심혈관질환의 발생 위험이 높다[3]. 또한 최근 10년 동안 성인 혈우병 환자의 비만률이 20% 이상 증가하면서[4] 혈우병 환자들에게 동반되는 심혈관질환 뿐 아니라 고혈압, 당뇨, 이상지질혈증과 같은 만성질환 관리의 중요성이 부각되고 있다.

혈우병 환자에게 통증과 기능부전을 유발하는 출혈은 대부분 외상, 무리한 근육의 사용, 스트레스 과다 등으로 인해 발생하고 출혈이 반복된다면 신체 기능 장애와 같은 합병증이 초래된다[5]. 이에 과거 혈우병 환자들은 신체활동과 운동을 제한하고 일상생활에서 출혈이 발생하지 않도록 조심할 것을 권고받았다[6]. 그러나 응고인자 예방요법이 도입되면서 혈우병 환자에게 심혈관질환이나 비만을 예방하고 관리하기 위해 신체활동과 운동을 권장하고 있다[7]. 혈우병 환자를 대상으로 한 선행연구에서 운동은 혈우병 환자의 통증[A1,A5,A8,A9]과 출혈 빈도[A1]를 감소시키며, 관절건강상태[A7,A8,A9,A10,A11], 관절기능[A2], 근력[A1,A3,A7,A8], 균형[A3,A8], 보행능력[A3], 호흡기능[A12], 신체활동[A7,A10,A11]을 증진시키는 신체적인 측면뿐만 아니라 우울, 불안, 자존감과 사회성, 협동심 등 심리사회적 측면[A13,^7^]과 의료비용 등 경제적인 측면[A6]에도 영향을 주며 궁극적으로는 삶의 질[A5,A6,A10,A12,A13]을 향상시키는 것으로 알려져 있다. 특히 근력강화운동은 염증세포 수를 감소시키고, 출혈 및 통증을 감소시키거나 예방하여 만성 관절병증이 있는 환자의 신체 기능을 회복시키는 것으로 보고되고 있다[8]. 또한 운동은 염증성 사이토카인[interleukin 6 (IL6), PCR, tumor necrosis factor-alpha (TNF-a)][9]의 농도를 감소시키며, 환자의 기동성, 근력, 조정력 및 신체 능력을 향상시키는 것으로 보고되어 있다[A7]. 저항 훈련은 중증 혈우병 B와 경증 또는 중등도 혈우병 A에서 응고인자 VIII 수준을 증가시키는 것으로 보고되고 있고[A4] 적절한 예방 조치를 취한다면 고충격 스포츠에 참여해도 출혈 위험이 증가하지 않는다고 보고되었다[A2]. 반면 운동이 출혈 위험을 증가시키거나 출혈 패턴에 변화를 유도한다는 연구 결과는 보고되지 않았다[10].

Nieva [11]는 혈우병 환자들은 운동의 긍정적인 효과에 대해 알고 있음에도 불구하고 언제 출혈이 발생할지 모르는 두려움, 관절 부위가 손상될 경우 관절 주위의 근육이 굳거나 통증을 유발하는 등 근육장애의 발생, 연령이 증가하면서 간병인들의 과잉보호와 혈우병 환자는 어떻게 운동해야 하는지에 대한 명확한 지침이 없어서 운동에 참여하지 않는다고 보고하였다.

이에 본 연구에서는 혈우병 환자들에게 실시한 운동 중재의 특성과 운동이 혈우병 환자들의 신체·생리적 변수, 심리·인지적 변수와 삶의 질에 미치는 효과를 파악하여 혈우병 환자들에게 운동을 교육할 때 활용할 수 있는 지침을 개발하는데 근거자료로 활용하고자 한다.

### 2. 연구의 목적

본 연구의 목적은 성인 혈우병 환자를 대상으로 운동의 효과를 검증한 무작위 대조군 실험연구(randomized controlled trial, RCT)를 체계적으로 고찰하여 운동 중재의 특성을 확인하고 운동 중재의 효과를 파악하는 것이다.

## 연구 방법

### 1. 연구 설계

본 연구는 성인 혈우병 환자에게 운동 중재를 실시한 RCT를 대상으로 운동 중재의 특성과 운동 중재의 효과를 파악하기 위해 체계적 문헌고찰과 메타분석을 실시한 연구이다.

### 2. 핵심 질문

핵심 질문 중 대상자는 혈우병을 진단받은 18세 이상인 자이고, 중재는 혈우병 환자에게 적용한 운동을 중재로 선정하였으며 운동의 형태는 유산소운동(aerobic training, AT), 근력강화운동(muscle strength training, MST), 저항운동(resistance training, RT) 및 기타운동을 모두 포함하였다. 문헌을 선정하기 위한 핵심 질문에서 비교중재와 결과변수는 제한하지 않았다

### 3. 문헌 검색 및 선정

#### 1) 문헌 검색

본 연구에서 문헌 검색은 2023년 3월 15일에 수행하였으며, 관련된 문헌을 모두 검색하기 위해 2023년 3월 14일까지 국내·외 학술지에 게재된 논문을 2명의 연구자가 독립적으로 2회 검색하였다. 검색 데이터베이스는 한국보건의료연구원(National Evidence-based Healthcare Collaborating Agency)이 제시한 국내·외 핵심 데이터베이스[12]를 참조하여 선정하였다. 국내에서 출판된 문헌은 한국교육학술정보원(Research Information Sharing Service, RISS), 의과학연구정보센터(Korean Medical Database, KMBASE)의 데이터베이스에서 검색하였다. 선행연구의 자료를 체계적으로 검색하기 위한 검색어는 PubMed와 Cochrane Library에서는 MeSH 용어를 활용하였고, Cumulative Index to Nursing and Allied Health Literature (CINAHL)에서는 CINAHL Headings을 활용하였으며, 국내 데이터베이스인 RISS, KMBASE의 경우 주요어를 조합하여 활용하였다. 또한 관련된 자연어를 추가하였고, 검색어간 불리언 연산자(AND, OR, NOT)를 조합하여 검색식으로 변환 후 검색하였다. 검색식은 (1) “혈우병” OR “응고인자 결핍” OR “응고인자 부족”, (2) “운동” OR "유산소운동" OR “근력강화” OR “저항훈련”으로 입력하여 (1)과 (2)를 연산자 ‘AND’로 검색식을 연결하였다. 국외는 PubMed, CINAHL, Cochrane library를 이용하여 검색하였다. 검색식은 (1) “Hemophilia” OR “Hemophilia A” OR “Hemophilia B” OR “von Willebrand Diseases” OR “Factor XI Deficiency” OR “Factor VIII deficiency” OR “Factor 8 Deficiency” OR “Factor 9 deficiency”, “Exercise” OR “Aerobic Training” OR “Muscle Strength Training” OR “Resistance Training” OR “weight training”으로 입력하고 (1)과 (2)를 연산자 ‘AND’로 검색식을 연결하였다.

#### 2) 문헌 선정

본 연구에서 문헌 선정은 PRISMA 그룹이 제시한 체계적 문헌고찰 지침[13]에 따라 수행하였으며, 문헌의 포함 기준과 배제 기준에 부합하는 문헌을 선정하였다.

##### (1) 포함 기준

연구 설계는 RCT만 포함하였다.

##### (2) 배제 기준

본 연구에서 문헌의 배제 기준은 혈우병 환자 중 정신질환이 있거나, 한국어 또는 영어 이외의 언어로 출판된 문헌, 원문(full text)을 검색할 수 없는 문헌이었다.

**Figure 1.**
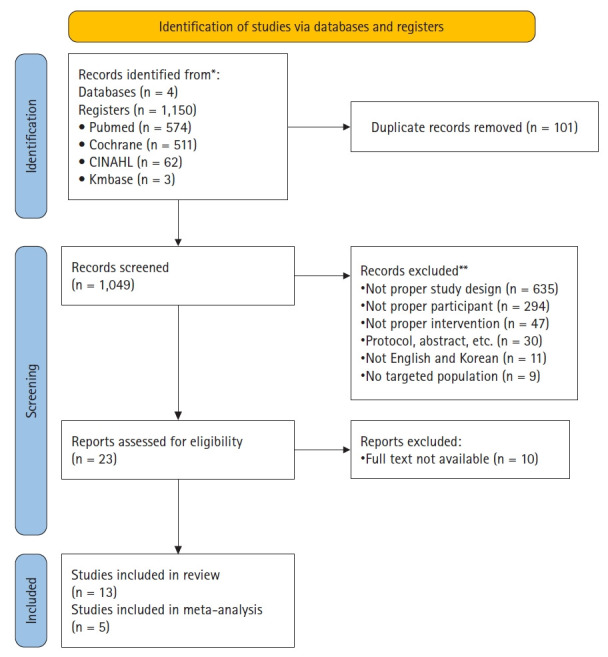
Flow chart of systematic review of literature selection process for the present study.

본 연구에서 국내·외 데이터베이스를 통해 검색된 문헌들은 EndNote X20, Microsoft Excel 2020 프로그램을 사용하여 분류하였다. 데이터베이스 검색결과 총 1,150편의 문헌이 검색되었다. 이중 중복된 101편을 제외한 1,049편의 문헌을 대상으로 제목, 초록을 검토하여 1차 선정하였으며 제목이나 초록을 통해서 확인이 어려운 문헌은 연구방법을 검토하여 문헌 선정 기준에 적합한 23편의 문헌을 선별하였다. 선별된 문헌 중에서 원문을 확인할 수 없는 10편을 제외하고 최종 13편의 문헌을 체계적 문헌고찰을 위한 질 평가 대상 문헌으로 선정하였다. 국내·외 데이터베이스를 통해 검색된 문헌검토와 선정은 2명의 연구자가 독립적으로 실시하였고, 연구자 간의 의견 불일치가 발생한 경우 문헌의 질 평가에 참여한 제3의 연구자가 다시 한번 평가하여 최종 선정하였다(Figure 1).

### 4. 자료 추출

체계적 문헌고찰을 시행하기 위해 선정된 대상 문헌의 저자, 출판연도, 연구대상자의 연령, 체질량지수(body mass index, BMI), 운동 중재 특성(운동의 종류, 기간, 빈도, 1회 시간, 평가도구, 평가결과 등)을 추출하여 Microsoft Excel 2020 프로그램을 사용하여 기록하였다. 연구자 1명이 이에 대한 서식을 작성하고, 추출한 내용의 정확성을 확보하기 위하여 연구자 2명이 독립적으로 분석한 후 교차 검토하였다. 운동 중재의 효과크기를 확인하고 연구에서 공통되는 결과지표의 값을 분석하기 위해 R program의 ‘Meta’ package을 사용하였다. 총 13편의 대상 문헌에서 메타분석을 실시한 결과지표는 ‘통증’, ‘관절건강상태’, ‘신체활동’, ‘삶의 질’이었다. 13편 중 메타분석이 가능한 문헌은 ‘통증’ 3편, ‘관절건강’ 4편, ‘신체활동’ 3편, ‘삶의 질’이 4편으로 총 8편이었다.

### 5. 자료 분석

#### 1) 문헌의 질 평가

본 연구에서는 RCT만을 대상으로 선정하였기 때문에 방법론적 질 평가는 Cochrane Risk of Bias 2.0 tool (RoB 2.0)[12]을 이용하여 평가하였다. RoB 2.0는 무작위 배정 과정에서 발생하는 비뚤림(bias arising from the randomisation process), 의도한 중재에서 이탈로 인한 비뚤림(bias due to deviations from the intended interventions), 중재결과 자료의 결측으로 인한 비뚤림(bias due to missing outcome data), 결과측정 비뚤림(bias in measurement of the outcome), 보고된 결과의 선택 비뚤림(bias in selection of the reported result)의 5개 항목을 평가하고, 이에 대한 전반적인 비뚤림 위험 평가(overall bias)를 실시하였다. 각 문항별로 비뚤림 위험을 ‘그렇다’, ‘아마도 그렇다’, ‘아마도 아니다’, ‘아니다’, ‘정보 없음’의 다섯 단계로 평가하며, 각 영역은 최종적으로 영역별 평가 알고리즘에 따라 ‘낮은 위험’, ‘일부 우려’, ‘높은 위험’의 세 단계로 판정하였다.

본 연구에서 문헌의 질 평가는 연구자 3명이 독립적으로 실시한 결과를 종합하여 평가자 간 의견이 일치하지 않는 경우 논의를 통해 합의점을 도출하였으며, 비뚤림 위험 가능성을 ‘높은 위험’, ‘낮은 위험’, ‘일부 우려’로 판단하였다.

#### 2) 효과크기와 이질성 분석

운동 중재의 효과크기 비교를 위해 R program의 ‘Meta’ package를 사용하여 메타분석을 시행하였다. 개별연구에서 추출된 운동 중재 관련 특성과 운동 효과와 관련된 결과변수의 값은 실험 처치 후의 평균과 표준편차를 이용하였고 각각의 실험결과를 표준화하기 위하여 표준화된 평균차이(standardized mean differences, SMD)를 이용하여 확인하였다. 사전에 선별된 개별연구 간의 연구 대상자, 운동 중재와 결과 변수의 측정 도구 간에 이질성이 존재한다고 판단되어 하나의 치료 효과 크기를 가정하기 어려운 경우이므로 변량효과모형(random effects model)으로 분석하였다[14]. 본 연구에서 효과 크기는 운동 중재 방법이 다양하고, 운동 효과에 대해 서로 다른 측정 도구를 사용하였기 때문에 연구 결과를 표준화하기 위해 SMD의 방법을 사용하여 확인하였다[12].

효과 크기의 해석은 전체 효과 크기가 0.2는 작은 효과 크기, 0.5는 중간 효과 크기, 0.8은 큰 효과 크기로 판단하였다[15]. 이질성 여부를 판단하기 위해 시각적 검토(forest-plot)를 통해 신뢰구간과 효과 값의 방향이 겹치는 부분이 있는지 확인하고, 비일관성을 정량화시킨 통계량인 통계적 검토(Higgin’s I_2_ 통계량)를 통해 이질성을 평가하였다. I_2_는 총 효과 크기의 분산에 대한 실제 분산의 비율을 의미하며, 이질성이 ‘25% 미만은 낮음’, ‘30%~60%는 중간’, ‘75% 이상은 매우 높음’으로 해석하였다[16].

#### 3) 출판 비뚤림

최종적으로 메타분석에 사용한 문헌들의 출판편향을 파악하기 위해 R package ‘metafor’(designed by Jeehyoung Kim in Seoul Sacred Heart General Hospital)로 만든 깔대기 도표(trimmed funnel plot)를 사용하여 검정하였다. 깔대기 도표는 가운데 기준을 중심으로 시각적으로 대칭 정도를 시각적으로 확인하고 효과 크기와 표준오차의 비대칭을 확인하기 위해 Egger’s 회귀분석을 실시하여 출판 삐뚤림이 메타분석 해석에 큰 영향을 미치지 않았다고 해석하였다[17].

## 연구 결과

### 1. 분석 대상 문헌의 일반적 특성

본 연구에서 분석한 문헌은 총 13편이며, 분석 대상 문헌의 일반적 특성은 Table 1과 같다. 문헌의 출판연도는 2014년부터 2023년까지이며, 2020년 이후 출판된 문헌이 총 6편이었다. 대상자의 성별은 모든 문헌에서 남성이었으며, 연구 대상자 수는 20∼64명, 평균연령은 40대가 7편으로 가장 많았고, 그 다음으로 30대가 4편이었다. BMI를 제시한 문헌이 9편이었으며 이중 8편에서 평균 BMI가 25.00∼29.99 kg/m^2^로 과체중이었다(Table 1).

### 2. 분석 대상 문헌의 질 평가

**Figure 2.**
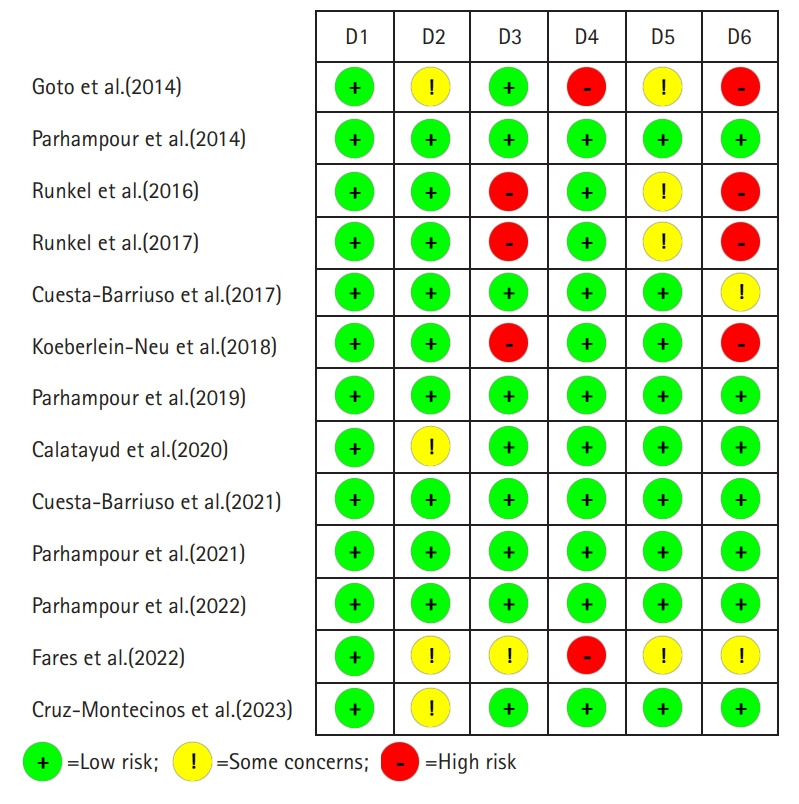
Risk of bias. D1 = Randomisation process; D2 = Deviations from the intended interventions; D3 = Missing outcome data; D4 = Measurement of the outcome; D5 = Selection of the reported result; D6 = Overall.

본 연구에서 최종 선택된 13편의 문헌의 질 평가 결과는 Figure 2와 같았다. 문헌의 질 평가 결과, 무작위 배정과정에서 발생하는 비뚤림 위험은 13편(100.0%)이 ‘낮은 비뚤림 위험’이었으며, 의도한 중재에서 이탈로 인한 비뚤림은 9편(69.2%)이 ‘낮은 비뚤림 위험’, 4편(30.8%)이 ‘일부 우려’이었다. 중재 결과 자료의 결측으로 인한 비뚤림은 9편(69.2%)이 ‘낮은 비뚤림 위험’, 3편(23.1%)이 ‘일부 우려’, 1편(7.7%)이 ‘높은 위험’으로 평가되었으며, 결과측정 비뚤림은 11편(84.6%)이 ‘낮은 비뚤림 위험’, 2편(15.4%)이 ‘높은 비뚤림 위험’으로 평가되었다. 보고된 결과 선택의 비뚤림은 9편(69.2%)이 ‘낮은 비뚤림 위험’, 4편(30.8%)이 ‘일부 우려’이었다. 전반적 문헌의 질 평가 결과 5가지 영역에서 최소 한 가지 이상에서 높은 비뚤림 위험이 있는 것으로 판정된 연구는 5편(38.5%)으로 평가되었으나 전반적으로 비뚤림 위험이 낮은 것으로 판정되어 모든 연구를 분석 대상에 포함시켰다(Figure 2).

### 3. 운동 중재의 특성

혈우병 환자를 위한 운동 중재에서 운동 종류, 운동 기간, 빈도, 시간은 Table 2와 같다. 운동 중재의 운동 종류는 RT와 복합운동(combined training, CT), AT를 비교한 연구 2편[A7,A10], RT와 CT를 비교한 연구 1편[A11], RT와 전기자극(pulsed electromagnetic field)을 함께 중재한 연구 1편[A1], RT 2편[A8,A9], 프로그램화된 스포츠 훈련(programmed sports therapy, PST) 2편[A2,A3], PST와 가정운동(home training, HT) 1편[A13], HT 2편[A5,A11], 물리치료 (physical therapy, PT)와 AT를 비교한 연구 1편[A12], 도수치료(manual therapy, MT)와 수동적 근육스트레칭(passive muscle stretch) 1편[A9]이었다. 혈우병 환자를 위한 운동 중재는 CT가 6편(46.2%)로 가장 많았으며 AT, HT, PST가 각 3편(23.1%), PT와 MT가 각 1편(7.7%) 순이었다(Table 2).

운동 중재의 운동 강도를 운동 기간, 운동 빈도, 운동 시간을 통해 살펴보면, 운동 기간은 6주에서 24주까지이며, 8주[A1,A8,A12,A13]와 6주[A1,A7,A10,A11]가 각 4편(30.8%)으로 가장 많았으며, 다음은 24주가 3편(23.1%)[A2,A3,A13], 15주[A5]와 12주[A9]가 각 1편(7.7%)이었다. 운동 빈도는 가정에서 하는 훈련으로 1주일에 7일을 실시한 연구가 2편(15.4%) 있었으며, 3회와 2회가 각 5편(38.5%), 1회 1편(7.7%) 순이었다. 운동 시간에 대해서는 4편의 연구에서 서술되어지지 않았으며, 30분 3편(23.1%), 30-40분 1편(7.7%), 40분 1편(7.7%), 45분 2편, 60분 1편(7.7%)이었다(Table 2).

### 4. 운동 중재의 효과

혈우병 환자를 위한 운동 중재에서 결과변수는 신체·생리적 변수, 심리·사회적 변수, 삶의 질로 분류되었다. 결과변수를 평가하는 도구는 혈액검사, 생리적 측정방법과 자가보고형 설문지가 사용되었다(Table 2). 신체·생리적 변수 중 근력, 균형, 보행능력, 관절가동범위, 면역지표, 염증 지표, 호흡기능 등은 생리적 측정방법을 이용하여 측정되었으며, 출혈 빈도, 통증, 관절건강, 관절기능, 신체활동은 자가보고형 설문지로 측정되었다.

본 연구에서 분석한 문헌 중 신체적 변수를 측정한 문헌은 체구성 3편[A7,A10,A11], 근력 4편[A1,A3,A7,A8], 균형 2편[A2,A8], 보행능력 1편[A2], 관절건강 5편[A7,A9,A8,A10,A11], 호흡기능 1편[A12], 관절기능 1편[A2], 통증 4편[A1,A5,A8,A9], 출혈 2편[A1,A9], 신체활동 3편[A7,A10,A11]이었다. 본 연구에서 분석한 문헌 중 신체적 변수를 측정한 문헌에서 호흡기능은 폐기능[A12]을, 출혈은 출혈 횟수[A1], 혈관절증 발생빈도[A9]를 결과변수로 평가하였다. 관절 기능은 자가보고형 설문지를 이용하여 측정되었다. 관절기능을 측정한 설문지는 수정된 콜로라도 설문지(modified colorado questionnaire)를 사용하였으며, 관절건강은 5편 모두 혈우병 환자의 관절건강 점수(hemophilia joint health score, HJHS) 측정 도구를 사용하였다. 신체활동은 자가보고형 설문지인 지각된 기능적 능력(perceived functional ability) 도구를 사용하여 측정하였으며 신체활동정도 1편[A1], 신체수행능력 1편[A3], 신체활동 4편[A7,A10,A11,A13]에서 결과 변수로 평가하였다. 통증은 4편[A1,A5,A8,A9]의 연구에서 시각적상사척도를 이용하여 측정하였다.

본 연구에서 분석한 문헌 중 생리적 변수를 측정한 문헌은 면역지표[A1], 염증지표 1편[A7]이었다. 분석한 문헌에서 측정한 생리적 지표는 Bone-specific ALP, N-terminal telopeptide of type 1 collagen, interleukin‐10, adiponectin, TNF‐α, interleukin‐6, CRP이었다.

본 연구에서 분석한 문헌에서 운동 효과에 대한 심리·사회적 변수 중 심리적 변수는 운동공포(kinesiophobia), 정서적 기능, 정신건강, 사회적 관계로 평가되었다. 운동공포는 1편[A8]의 문헌에서, 정서적 기능과 정신건강, 사회적 관계도 1편[A6]의 문헌에서 평가하였다. 본 연구에서 분석한 문헌에서 운동 효과에 대한 인지적 변수로는 질병행동, 주관적 건강상태, 운동 욕구 등으로 평가되었다. 인지적 변수 중 질병행동[A5], 주관적 건강상태[A8], 운동 욕구[A8]는 각 1편의 문헌에서 평가하였다.

본 연구의 문헌에서 운동 효과에 대해 삶의 질을 결과변수로 측정한 문헌은 5편[A4,A5,A6,A10,A13]이었다. 삶의 질을 측정한 도구는 일반적인 삶의 질을 측정하는 도구(Euro quality of life Questionnaire 5-Dimensional Classification, EQ-5D), 건강관련 삶의 질을 측정하는 도구(Short Form-36, SF-36), 혈우병 환자의 삶의 질을 측정하는 도구(haemophilia specific quality of life, Haem-A-QOL)로 측정되었다.

### 5. 효과 크기와 이질성 검정

본 연구에서 운동 중재의 효과크기를 산출하기 위해 실험군과 대조군의 사전, 사후 평균과 표준편차 값을 제시한 문헌만 메타분석에 포함시켰다.

#### 1) 통증

**Figure 3.**
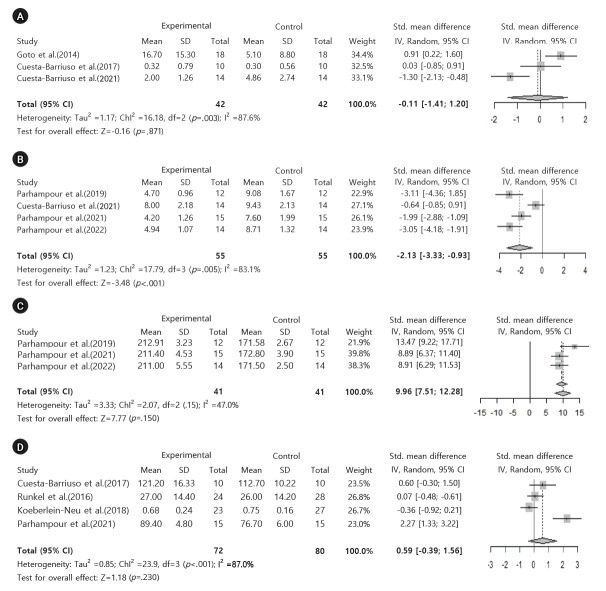
Forest plots of the effects of exercise. (A) Forest plot of the effects of exercise on pain. (B) Forest plot ot the effects of exercise on joint health. (C) Forest plot of the effects of exercise on physical activity. (D) Forest plot of the effects of exercise on quality of life. SD = standard deviation; CI = confidence interval.

본 연구에서 분석한 문헌 중 통증은 4편[A1,A5,A8,A9]의 연구에서 결과를 보고하였으며 3편의 연구[A5,A8,A9]에서는 유의하게 감소하였으나 1편의 연구[A1]에서는 유의한 차이가 없었다. 운동 중재가 통증에 미치는 효과크기를 산출하기 위해 통증의 값을 보고한 문헌 4편 중 3편의 연구[A1,A5,A9]에 대해 메타분석을 실시하였다. 3편의 연구에서 통증은 시각적 상사척도(visual analogue scale)로 측정하였으며, 이 도구의 점수범위는 0∼10점이며 점수가 낮을수록 통증이 낮다는 것을 의미한다. 연구의 이질성 검정 결과 이질성이 87.6%로 큰 크기의 이질성이 확인되어 변량효과모형을 사용하여 분석하였다. 전체 연구의 SMD는 –0.11 (95% confidence interval [CI] = -1.41∼1.20)로 통계적으로 유의하지 않았다(Z = -0.16, p = .871) (Figure 3A).

#### 2) 관절건강

본 연구에서 운동중재가 관절건강에 미치는 효과크기를 산출하기 위해 관절건강의 값을 보고한 문헌 4편을 대상으로 메타분석을 실시하였다. 4편의 연구에서 관절건강은 혈우병환자 HJHS로 측정하였으며 이 도구의 점수범위는 0∼124점이며 점수가 낮을수록 관절건강상태가 양호함을 의미한다. 연구의 이질성 검정 결과 이질성이 83.1%로 큰 크기의 이질성이 확인되어 변량효과모형을 사용하여 분석하였다. 관절건강에 대해 보고한 4편의 SMD는 –2.13 (95% CI = -3.33, -0.93)으로 관절건강이 증가하는 효과가 있었으며 통계적으로 유의한 차이가 있었다(Z = -3.48, *p* < .001) (Figure 3B).

#### 3) 신체활동

본 연구에서 운동중재가 신체활동에 미치는 효과크기를 산출하기 위해 신체활동의 값을 보고한 문헌 3편을 대상으로 메타분석을 실시하였다. 3편의 연구에서 신체활동은 혈우병환자 활동 리스트 설문지(haemophilia activities list questionnaire)로 측정하였다. 이 도구는 42개 문항 6점 Likert 척도로 점수범위는 42∼252점이며 점수가 높을수록 신체활동이 높음을 의미한다. 연구의 이질성 검정 결과 이질성이 83.1%로 큰 크기의 이질성이 확인되어 변량효과모형을 사용하여 분석하였다. 신체활동에 대해 보고한 3편의 SMD는 9.96 (95% CI = 7.51, 12.28)으로 신체활동이 증가하는 효과가 있었으며 통계적으로 유의한 차이가 있었다(Z = 7.77, *p* < .001) (Figure 3C).

#### 4) 삶의 질

본 연구에서 운동 중재가 삶의 질에 미치는 효과크기를 산출하기 위해 삶의 질을 보고한 문헌 5편의 연구 중에 삶의 질 값을 보고한 4편을 대상으로 메타분석을 실시하였다. 4편의 연구 중 1편은 혈우병 환자 특이 삶의 질 도구인 A36 Hemophilia-QoL®로, 1편은 SF-36, 1편은 EQ-5D로 측정하였으며, 1편의 연구는 SF-36과 A36 Hemofilia-QoL®로 삶의 질을 측정하였다. A36 Hemofilia-QoL®은 36개 문항 5점 Likert 척도로 점수 범위는 36∼180점이며 점수가 낮을수록 삶의 질이 높음을 의미한다. 3편[A4,A5,A12]의 문헌에서는 운동 중재 후에 삶의 질이 유의하게 향상되었다고 보고하였다. SMD는 0.59 (95% CI = -0.39, 1.56)로 통계적으로 유의한 차이는 없었다(Z = 1.18, *p* = .230) (Figure 3D).

## 논의

본 연구는 성인 혈우병 환자를 대상으로 운동 중재를 실시한 RCT 연구를 분석하여 운동 중재의 특성과 효과를 파악하기 위한 체계적 문헌고찰 연구이다. 본 연구에서는 문헌 선정 기준에 적합한 13편의 연구를 분석하였다. 선정된 연구 중 국외 연구는 일본 1편, 독일 3편, 이란 3편, 스페인 4편, 이집트 1편이었고, 국내 연구는 없었다. 한국혈우재단[18]의 보고에 의하면 2019년 현재 한국혈우재단에 Ⅷ인자결핍 혈우병A는 1,746명(69.6%), Ⅸ인자결핍 혈우병B는 434명(17.3%)으로 총 2,509명이 등록되어 있다. 이는 추후 국내에서도 성인 혈우병 환자를 대상으로 한 운동 중재를 시행하는 연구가 필요함을 시사한다.

선택된 13개의 연구에서 운동 중재의 특성을 분석한 결과, 운동의 종류는 MST와 AT를 함께 실시하는 CT가 가장 많았으며, 그 외에 RT, AT, HT, 전기자극, 물리치료 등이 있었다. 그 중 9편은 운동의 종류에 따른 효과를 비교하기 위해 2개 이상의 대조군에서 서로 다른 운동을 실시하고 그 효과를 비교 분석한 연구이었다. 운동의 처방을 확인하기 위해 운동 기간, 빈도, 시간과 강도를 분석하고자 하였으나 운동 강도를 제시한 연구가 없어서 운동 기간, 빈도, 시간만을 분석하였다. 본 연구에서 분석한 연구들의 운동 기간은 6주에서 24주까지 매우 다양하였으며, 운동 빈도는 가정 운동의 경우는 1주일에 6∼7회이었고 그 외는 1주일에 1회∼3회이었고, 운동 시간은 30분에서 60분까지이었다. 본 연구 결과를 근거로 현재 혈우병 환자들의 특성을 고려한 운동처방(운동의 종류, 기간, 빈도, 시간, 강도)에 대해 표준화된 공식 지침이 없음을 알 수 있었다. 이는 혈우병 환자들은 운동 지침이 명확하지 않기 때문에 어떻게 해야 할지 몰라서 운동을 하지 않는다고 보고한 Nieva의 연구[11]의 결과와 일치한다.

세계혈우병재단(World Federation of Hemophilia)은 2006년 혈우병 환자를 위한 재활운동지침[19]에서 혈우병성 출혈이 많이 발생하는 무릎, 발목과 팔꿈치 관절의 기능을 강화시킬 수 있는 근력 강화운동을 소개하였다. 2020년에는 혈우병 환자관리 지침[20]에 혈우병 환자 중 근골격계 장애가 있으면 관절가동 범위 내에서 골밀도를 높일 수 있는 체중부하 운동을 권장하였으며, 수영, 걷기, 조깅, 골프, 배드민턴, 양궁, 자전거 타기, 조정, 항해, 탁구 등 비접촉성 스포츠는 권장하나 축구, 하키, 럭비, 복싱, 레슬링 등 접촉이 많고 충돌이 많은 스포츠와 모터크로스 경주, 스키 등 고속 활동은 생명을 위협하는 부상을 입을 수 있으므로 권장되지 않는다. 그러나 2006년은 관절가동범위를 증진시킬 수 있는 근력강화운동만, 2020년에는 혈우병 환자의 운동과 관련된 전반적인 안내만 이루어졌기 때문에 혈우병 환자들이 일상생활에서 적용하기는 부적합하다고 생각된다. 추후 혈우병 환자를 위한 운동 지침을 개발할 때는 환자의 신체적, 기능적, 심리적, 사회문화적 환경을 고려할 것과, 운동의 형태와 강도, 빈도 등을 제시한 구체적인 신체활동지침을 개발하고 권고해야 할 필요가 있다.

본 연구에서 혈우병 환자를 위한 운동 중재의 효과는 신체·생리적 변수, 심리·사회적 변수와 삶의 질로 분류되며 신체·생리적 변수는 신체활동 정도, 체구성, 근력, 균형, 보행능력, 관절가동범위, 관절기능, 관절건강, 통증, 면역인자, 염증인자, 출혈 빈도 등이 평가되어졌으며 심리·사회적 변수는 운동 공포, 정서적 기능, 정신건강, 사회적 관계가 평가되어졌고 삶의 질은 전반적인 삶의 질, 건강관련 삶의 질, 혈우병관련 삶의 질 등으로 평가되었다. 그러나 본 연구에서 분석된 문헌의 심리·사회적 변수는 측정 항목이나 도구가 일치하지 않아 메타분석에 포함되지 못하였다. 추후 운동의 신체생리적 효과뿐 아니라 심리사회적 변수의 효과에 대해서도 검증할 것을 제안한다.

본 연구는 성인 혈우병 환자의 운동 중재의 효과를 검증하기 위해 메타분석을 실시하였다. 선정된 연구 중 통증은 3편의 연구, 관절건강은 4편, 신체활동 3편, 삶의 질 4편을 대상으로 메타분석을 통해 효과크기를 검정하였다. 본 연구에서 통증은 유의하게 감소하지 않은 것으로 나타났다. 그러나 혈우병 환자의 운동 중재의 효과에 대해 체계적 고찰한 Schäfer 등[21]은 2개 연구에서는 통증이 감소하였으나 1개 연구에서는 감소하지 않은 것으로 보고하였고, 또 다른 체계적 고찰 선행 연구인 Chen, Lin과 Kung [22]의 연구에서는 통증이 유의하게 감소하였다고 보고하였다. 본 연구에서 통증이 유의하게 감소하지 않은 2개 연구는 HT였고, 통증이 감소한 문헌에서 수행한 운동은 물리치료로 근력강화 재활운동을 실시한 연구였다. 운동은 근육을 활성화시켜 혈액 순환을 촉진시키며, 이는 관절에 더 효율적으로 산소를 공급하여[23,24] 통증을 감소시킨 것으로 판단한다. 그러나 과도한 운동은 근육의 통증과 출혈을 유발할 수 있으며 HT를 실시할 때는 적절한 휴식을 취하면서 운동하는 것이 필요하다[25]. 또한 출혈의 위험이 높다면 진단 초음파를 통해 출혈이나 혈종의 진행과정을 모니터링하면서 의료인의 지시에 따라 운동할 것을 권장한다[25]. 최근 4차 산업혁명으로 정보통신 기술이 발달하고 있으므로 혈우병 환자들에게도 사물인터넷을 기반으로 웨어러블 기기나 스마트폰을 활용하여 대상자의 운동과 건강상태를 모니터링하고, 정보를 공유할 수 있는 장치를 활용할 것을 제안한다.

본 연구에서는 자가설문지로 측정된 관절건강과 신체활동이 향상된 것으로 확인되었다. 이는 Chen 등[22]이 혈우병 환자의 운동을 포함한 물리치료에서 관절가동범위와 관절건강이 향상되었다고 보고한 결과와 일치한다. 이는 운동이 혈우병 환자들의 출혈로 인한 손상을 회복하는데 도움이 되었을 뿐 아니라 근육이 굳거나 통증을 유발하는 근육장애를 감소시켰기 때문으로 판단된다. 본 연구 결과를 근거로 운동 중재를 성인 혈우병 환자의 관절기능과 신체활동을 증진시키는 중재로 활용할 것을 제안한다. Jin과 Kim [26]은 심부전 환자가 운동을 실천하는데는 운동에 대한 정보, 개인적 동기, 행동기술(자기효능감), 우울이 영향을 미친다고 보고하였으며 Kim과 Lee [23]는 강직성 척추염 환자가 운동을 지속하는데 건강전문가의 자율성 지지, 기본심리욕구 만족도의 유능성 및 관계성, 자율적 동기화 과정이 영향을 미친다고 보고하였다. 이와 같은 선행연구 결과를 근거로 추후 성인 혈우병 환자의 운동 시작과 지속에 영향을 미치는 요인을 광범위하게 규명하고 이를 포함하는 중재를 개발하고 그 효과를 규명하는 연구를 제안한다. 본 연구는 영어로 보고된 연구만을 분석하였기 때문에 사회문화적 측면에서 연구결과를 해석하는데 제한점이 있으므로 성인 혈우병 환자의 운동중재를 다양한 지역의 국가와 환경에 있는 대상자에게 확대 적용하기 위해서는 체계적 문헌고찰에 영어 이외의 언어로 발표된 논문을 포함시킬 것을 제안한다.

## 결론

본 연구에서 성인 혈우병 환자의 운동 중재를 체계적으로 문헌고찰한 결과 운동의 종류는 CT가 가장 많이 수행되고 있었으며 그 외에 AT, HT, PST 등이 적용되고, 운동 기간은 6주에서 24주이었으며 24주가 가장 많았다. 운동 빈도는 HT는 1주 5∼6일, 그 외는 1주 2∼3일로 운동의 형태에 따라 다양하였다. 운동 중재는 성인 혈우병 환자의 관절건강과 신체활동을 증가시키는데 유의한 효과가 있는 것으로 확인되었다. 이를 근거로 성인 혈우병 환자는 AT와 MST를 함께 실시하는 CT를 적어도 1주일에 2일 이상 실시할 것을 제안한다. 그러나 본 연구에서 국내 데이터베이스를 검색한 결과 성인 혈우병 환자를 대상으로 운동 중재를 시행한 RCT는 검색되지 않았다. 본 연구 결과를 근거로 국내에서도 성인 혈우병 환자를 대상으로 한 운동 중재를 개발하고 이를 적용하여 그 효과를 규명하는 연구가 필요하다.

## Figures and Tables

**Table 1.** Characteristics of the Included Studies (N = 13)

No.	Author (yr)	Sample size (n)		Sex (M/F)		Age (yr)		BMI (kg/㎡)	
A1	Goto et al. (2014)	E	16	E	16/0	E	41.8	E	-
		C	16	C	16/0	C	43.9	C	-
A2	Parhampour et al. (2014)	E₁	13	E₁	13/0	E₁	-	E₁	23.4
		E₂	12	E₂	12/0	E₂	-	E₂	23.0
		E₃	11	E₃	11/0	E₃	-	E₃	23.0
		C	12	C	12/0	C	-	C	22.7
A3	Runkel et al. (2016)	E	24	E	24/0	E	41.9	E	26.8
		C	28	C	28/0	C	40.3	C	26.2
A4	Runkel et al. (2017)	E	24	E	24/0	E	41.9	E	-
		C	28	C	28/0	C	40.3	C	-
A5	Cuesta-Barriuso et al. (2017)	E	10	E	10/0	E	31.9	E	-
		C	10	C	10/0	C	30.0	C	-
A6	Koeberlein-Neu et al. (2018)	E	23	E	23/0	E	42.1	E	26.8
		C	27	C	27/0	C	40.2	C	26.2
A7	Parhampour et al. (2019)	E₁	12	E₁	12/0	E₁	46.4	E₁	27.7
		E₂	12	E₂	12/0	E₂ E₃ C	45.8	E₂ E₃	27.9
		E₃	12	E₃	12/0		45.5	C	27.7
		C	12	C	12/0		45.1		27.6
A8	Calatayud et al. (2020)	E	10	E	10/0	E	39.1	E	-
		C	10	C	10/0	C	36.3	C	-
A9	Cuesta-Barriuso et al. (2021)	E	14	E	14/0	E	32.5	E	26.8
		C	14	C	14/0	C	31.1	C	26.8
A10	Parhampour et al. (2021)	E₁	15	E₁	15/0	E₁	46.8	E₁	27.8
		E₂	15	E₂	15/0	E₂	46.2	E₂	27.7
		E₃	15	E₃	15/0	E₃	46.0	E₃	28.0
		C	15	C	15/0	C	46.0	C	27.8
A11	Parhampour et al. (2022)	E₁	14	E₁	14/0	E₁	46.7	E₁	27.8
		E₂	14	E₂	14/0	E₂	46.0	E₂	28.0
		C	14	C	14/0	C	45.7	C	27.8
A12	Fares et al. (2022)	E	20	E	20/0	E	28.3	E	28.1
		C	20	C	20/0	C	27.8	C	27.9
A13	Cruz-Montecinos et al. (2023)	E	10	E	10/0	E	39.1	E	27.0
		C	10	C	10/0	C	36.3	C	27.2

M = male; F = female; BMI = body mass index; E = experimental group; C = control group.

**Table 2.** Exercise Interventions and Outcome Variables of the Included Studies

No.	Author (yr)	Exercise program			Measurements	Outcome variables
		Type		Duration/frequency/time		
A1	Goto et al. (2014)	E	HT & self-monitoring program (feedback system and activity monitor)	8 weeks/7 days/1 session	· Physical function: strength of knee extension, PROM in knee and ankle, dynamic standing position balance, 10-meter walking time	· Physical function
		C	HT & undisplayed activity monitor		· Physical activity: METs	- Strength of knee extension, range of knee extension, range of ankle dorsiflexion, modified functional reach (+)
					· Bleeding history, injection of a coagulation factor	- 10-min gait time (-)
					· Pain: visual analogue scale	· Physical activity (NS)
					· Self-efficacy for exercise: questionnaire	· Bleeding tendency (NS)
					· Exercise adherence: questionnaire	· Pain (NS)
						· Exercise adherence (+)
						· Self-efficacy for exercise (+)
A2	Parhampour et al. (2014)	E₁	RT	6 week/3 times/ 30-40 min	· Biochemical markers: NTX, BALP, tALP, Ca, P	· E₁, E₂ group
		E₂	RT & PEMF		· Muscle strength: chest press, shoulder press, scapular retraction, hip flexion/extension /abduction and knee extension	- BALP(+), tALP(+),
		E₃	PEMF		· Joint function: modified Colorado questionnaire	- Muscle strength (+)
		C	No intervention			- Joint function: knee, ankle, elbow (+)
						· E₃: only knee joint function (+)
A3	Runkel et al. (2016)	E	PST	24 weeks/ 2 times a week/ 90 min	· Complex strength: isometric strength performance, 6 muscle	· Complex strength (muscle triceps brachii, biceps brachii, latissimus dorsi, rectus abdominis, biceps femoris, quadriceps femoris) (+)
		C	No intervention		· Walking capacity: 12-min walking test: distance covered within 12 min	· 12-min walking test: walking distance (+)
					· Balance: one leg stand: 30 seconds	· Balance: right leg (+)
					· Physical condition of joint: Gilbert scale questionnaire	· Physical condition of joint: right knee (+)
A4	Runkel et al. (2017)	E	PST	24 weeks/ 2 times a week/ 90 min	· Subjective physical performance: HEP test Q(Physical performance)	· Subjective physical performance
		C	No intervention		· QOL: SF-36, Haem-A QOL questionnaire	- strength & coordination, endurance, body perception(+)
						- HEP test Q total score (+)
						· QOL
						- SF-36: general health perception, mental health (+)
						- Haem-A QOL: feeling, work, family (+)
A5	Cuesta-Barriuso et al. (2017)	E	Educational intervention & HT	· Education: 12 weeks/ 1 times 2 weeks/ 60 min	· Joint deterioration: Pettersson scale	· Joint deterioration (-)
				· Home exercise: 15 weeks/ 6 days/1 session a day	· Physical condition of joint: Gilbert scale questionnaire	· Physical condition of joint (NS)
					· Pain: visual analogue scale	· Pain: ankle(-)
		C	No intervention		· Illness behavior: IBQ questionnaire	· Illness behavior
					· QOL: A36 Haem-A QOL questionnaire	- Hypochondriasis, psychological & somatic perception (-)
						· Haem-A QOL (+)
						- Physical health, daily activities, joints, pain, emotional functioning (+)
						- overall quality of life (+)
A6	Koeberlein-Neu et al. (2018)	E	PST & HT	24 weeks/ 2 times a week/ 90 min	· Resource utilization: questionnaire	· Quality-adjusted life years (+)
		C	Usual care		· Cost-effectiveness, acceptability curve, sensitivity analysis	· Cost-effectiveness (+)
					· Quality-adjusted life years (QALYs)	· QOL (+)
					· QOL: Euro QoL-5domain questionnaire	
A7	Parhampour et al. (2019)	E₁	RT	6 weeks/ 3 times a week/ 45 min	· Biochemical markers: interleukin‐10, adiponectin, TNF‐α, interleukin‐6, CRP	· E₁, E₂, E₃
		E₂	AT		· Anthropometric assessment: WC, WHR, fat mass, fat‐free mass(Watson formula)	- WC, WHR, BMI weight
		E₃	CT		· Haemophilia Activities List: HAL questionnaire	- Joint health status (-)
		C	No intervention		· Joint health status: HJHS questionnaire	- HAL (+), adiponectin (NS)
						· CT group: hsCRP, IL-6, TNF-α, IL-10 (-)
A8	Calatayud et al. (2020)	E	RT	8 weeks/2 times a week/ 3session	· Muscle strength: knee, ankle, elbow joint	· Muscle strength,
		C	No intervention		· Balance: timed “up and go” test score, sit-to-stand	- Elbow: all flexion & extension (+)
					· PROM	- Knee left flexion & all extension (+)
					· Joint health status: HJHS questionnaire	· Timed “up and go”, sit-to-stand scores (+)
					· Phobia: Kinesiophobia score questionnaire	· ROM, right knee flexion (+)
					· Pain: global impression of pain change	· Pain (-)
					· General self-rated health status: questionnaire	· HJHS (left knee) (-)
					· Desire to exercise: questionnaire	· Desire to exercise (+)
						· General self-rated health status(+)
						· Desire to exercise(+)
A9	Cuesta-Barriuso et al. (2021)	E	Manual therapy & passive muscle stretching	12 weeks/ 2 times a week/ 60 min	· Frequency of knee hemarthrosis: self-reporting	· Frequency of knee hemarthrosis, (-)
					· Range of motion: goniometry	· Range of motion in knee flexion (+)
					· Knee pain: visual analogue scale	· Loss of extension (-)
		C	No intervention		· Joint health status: HJHS questionnaire	· Pain (-)
						· Joint health status (-)
A10	Parhampour et al. (2021)	E₁	RT	6 weeks/ 3 times a week/ 45 min	· Lipid profile: TC, TG, LDL, HDL	· E₁, E₂, E₃
		E₂	AT		· Anthropometric assessment: WHR, WC	- Weight, BMI, WHR, WC (-),
		E₃	CT		· Joint health status: HJHS questionnaire	- Lipid profile: LDL, TC, TG (-) / HDL (+)
		C	Not described		· Perceived functional ability: HAL questionnaire	- Joint health status (-)
					· QOL: SF-36 questionnaire	- Perceived functional ability (+)
						- QOL (+)
A11	Parhampour et al. (2022)	E₁	RT	6 weeks/ 3 times a week / 40 min resistance or 20 min resistance + 20 min aerobic	· Anthropometric assessment(ultrasonography): MT, PA (BB, TB, VM, VL, MG)	· E₁, E₂
		E₂	CT		· Joint health status: HJHS questionnaire	- MT: 5 all muscles (+)
		C	No intervention		· Perceived functional ability: HAL questionnaire	- PA: 3 muscles (VM, VL, MG)(+)
						- Joint health status (-)
						- Perceived functional ability (+)
A12	Fares et al. (2022)	E	PT & AT	8 weeks/ 3 times a week / 30 min	· PFT: FVC, FEV1, FEV1/FVC, PEF, PIF, MVV	· PFT (+)
		C	PT			
A13	Cruz-Montecinos et al. (2023)	E	RT	8 weeks/ 2 times a week / 3 sessions	· Emotional functioning, mental health, social relationship: questionnaire	· Emotional functioning, mental health, social relationship (NS)
		C	No intervention		· Joint health status: HJHS questionnaire	· Joint health status (-)
					· Perceived functional ability: HAL questionnaire	· Perceived functional ability
					· QOL: A35 hemophilia-QOL questionnaire	- Lying, sitting, kneeing, standing (+)
						- Leisure activities and sports (+)
						- Complex lower extremity activity) (+)
						- Sum score (+)
						· QOL
						- Physical health, joints, pain, emotional functioning, overall quality of life (+)

AT = aerobic training group; BB = biceps brachii; BALP = bone-specific alkaline phosphatase; C = control group; Ca = calcium; CRP = C-reactive protein; CT = combined training group; E = experimental group; HAL = Hemophilia Activities List; HDL = high-density lipoprotein; Haem-A QOL = Haemophilia-specific QOL; HEP-Test-Q = Haemophilia & Exercise Project questionnaire; HJHS score = Hemophilia Joint Health Score; HOUSEH = household tasks; HT = home training; IBQ = Illness Behavior Questionnaire; LDL = low-density lipoprotein; LEISPO = leisure activities and sports; LSKS = lying/sitting/kneeling/standing; MT = muscle thickness; NS = not significant; NTX = N-terminal telopeptide of type 1 collagen; QALYs = quality-adjusted life years; QOL = quality of life; TC = total cholesterol; TB = triceps brachii; TG = triglyceride; TNF‐α = tumor necrosis factor‐α; PA = pennation angle; MG = medial gastrocnemius; P = phosphorus; PEMF = pulsed electromagnetic field; PFT = pulmonary function; PROM = passive range of motion; PST = programmed sports therapy; PT = physical therapy; RT = resistance training; RTPEMF = resistance training group & pulsed electromagnetic field; SELFC = self-care; tALP = total alkaline phosphatase; VL = vastus lateralis; VM = vastus medialis; WC = waist circumference; WHR = waist‐to‐hip ratio; (+) = experimental group significantly improved; (-) = experimental group significantly decreased.

## Data Availability

The data supporting the findings of this study are available in the article and appendix.

## Appendix 1. Studies included in the systematic review and meta-analysis

A1. Goto M, Takedani H, Haga N, Kubota M, Ishiyama M, Ito S, et al. Self‐monitoring has potential for home exercise programmes in patients with haemophilia. Haemophilia. 2014;20(2):e121-e127. https://doi.org/10.1111/hae.12355

A2. Parhampour B, Torkaman G, Hoorfar H, Hedayati M, Ravanbod R. Effects of short-term resistance training and pulsed electromagnetic fields on bone metabolism and joint function in severe haemophilia A patients with osteoporosis: a randomized controlled trial. Clinical Rehabilitation. 2014;28(5):440-450. https://doi.org/10.1177/0269215513505299

A3. Runkel B, Czepa D, Hilberg T. RCT of a 6‐month programmed sports therapy (PST) in patients with haemophilia–Improvement of physical fitness. Haemophilia. 2016;22(5):765-771. https://doi.org/10.1111/hae.12957

A4. Runkel B, Von Mackensen S, Hilberg T. RCT–subjective physical performance and quality of life after a 6‐month programmed sports therapy (PST) in patients with haemophilia. Haemophilia. 2017;23(1):144-151. https://doi.org/10.1111/hae.13079

A5. Cuesta-Barriuso R, Torres-Ortuño A, Nieto-Munuera J, López-Pina JA. Effectiveness of an educational physiotherapy and therapeutic exercise program in adult patients with hemophilia: a randomized controlled trial. Archives of Physical Medicine and Rehabilitation. 2017;98(5):841-848. https://doi.org/10.1016/j.apmr.2016.10.014

A6. Koeberlein‐Neu J, Runkel B, Hilberg T. Cost‐utility of a six‐month programmed sports therapy (PST) in patients with haemophilia. Haemophilia. 2018;24(3):385-394. https://doi.org/10.1111/hae.13459

A7. Parhampour B, Dadgoo M, Vasaghi‐Gharamaleki B, Torkaman G, Ravanbod R, Mirzaii-Dizgah I, et al. The effects of six‐week resistance, aerobic and combined exercises on the pro‐inflammatory and anti‐inflammatory markers in overweight patients with moderate haemophilia A: a randomized controlled trial. Haemophilia. 2019;25(4):e257-e266. https://doi.org/10.1111/hae.13764

A8. Calatayud J, Pérez-Alenda S, Carrasco JJ, Cruz-Montecinos C, Andersen LL, Bonanad S, et al. Safety and effectiveness of progressive moderate-to-vigorous intensity elastic resistance training on physical function and pain in people with hemophilia. Physical Therapy. 2020;100(9):1632-1644. https://doi.org/10.1093/ptj/pzaa106

A9. Cuesta‐Barriuso R, Gómez‐Conesa A, López‐Pina JA. The effectiveness of manual therapy in addition to passive stretching exercises in the treatment of patients with haemophilic knee arthropathy: a randomized, single‐blind clinical trial. Haemophilia. 2021;27(1):e110-e118. https://doi.org/10.1111/hae.14181

A10. Parhampour B, Dadgoo M, Torkaman G, Ravanbod R, Bahri TD, Jazebi M, et al. Effects of short-term aerobic, resistance and combined exercises on the lipid profiles and quality of life in overweight individuals with moderate hemophilia A: a randomized controlled trial. Medical Journal of the Islamic Republic of Iran. 2021;35(1):542-553. http://dx.doi.org/10.47176/mjiri.35.70

A11. Parhampour B, Alizadeh V, Torkaman G, Ravanbod R, Bagheri R, Vasaghi‐Gharamaleki B, et al. Muscle thickness and pennation angle in overweight persons with moderate haemophilia A after resistance and combined training: a randomized controlled trial. Haemophilia. 2022;28(3):505-514. https://doi.org/10.1111/hae.14539

A12. Fares HM, Ahmed SH, Farhat ES, Alshahrani MS, Abdelbasset WK. The efficacy of aerobic training on the pulmonary functions of hemophilic A patients: a randomized controlled trial. European Review for Medical and Pharmacological Sciences. 2022;26(11):3950-3957. https://doi.org/10.26355/eurrev_202206_28964

A13. Cruz-Montecinos C, Pérez-Alenda S, Casaña J, Carrasco JJ, Andersen LL, López-Bueno R, et al. Effectiveness of progressive moderate‐vigorous intensity elastic resistance training on quality of life and perceived functional abilities in people with hemophilia: secondary analysis of a randomized controlled trial. European Journal of Haematology. 2023;110(3):253-261. https://doi.org/10.1111/ejh.13900
